# Involvement of Th1Th17 Cell Subpopulations in the Immune Responses of Mothers Who Gave Birth to Children with Congenital Zika Syndrome (CZS)

**DOI:** 10.3390/v14020250

**Published:** 2022-01-26

**Authors:** Iury Amancio Paiva, Débora Familiar-Macedo, Jéssica Badolato-Corrêa, Fabiana Rabe Carvalho, Helver Gonçalves Dias, Alex Pauvolid-Corrêa, Caroline Fernandes dos Santos, Andréa Alice Silva, Elzinandes Leal de Azeredo, Renata Artimos de Oliveira Vianna, Claudete Aparecida Araújo Cardoso, Alba Grifoni, Alessandro Sette, Daniela Weiskopf, Luzia Maria de-Oliveira-Pinto

**Affiliations:** 1 Laboratory of Viral Immunology, Fundação Oswaldo Cruz, Rio de Janeiro 21040-360, Brazil; iury.iap@gmail.com (I.A.P.); deborafamiliar@gmail.com (D.F.-M.); jessicabadolato04@gmail.com (J.B.-C.); helvergd@gmail.com (H.G.D.); carol.uned@gmail.com (C.F.d.S.); naideazeredo@gmail.com (E.L.d.A.); 2 Multiuser Laboratory for Research in Nephrology and Medical Science, School of Medicine, Universidade Federal Fluminense, Niterói 24033-900, Brazil; fabiana.r.carvalho@hotmail.com (F.R.C.); aasilva@id.uff.br (A.A.S.); claudetecardoso@id.uff.br (C.A.A.C.); 3 Department of Veterinary Integrative Biosciences, Texas A&M University, College Station, TX 77843-4458, USA; pauvolid@gmail.com; 4 Laboratory of Respiratory Viruses and Measles, Fiocruz, Rio de Janeiro 21040-360, Brazil; 5 Department of Maternal and Child, School of Medicine, Universidade Federal Fluminense, Niterói 24033-900, Brazil; renatavianna03@gmail.com; 6 Center for Infectious Disease and Vaccine Research, La Jolla Institute for Immunology (LJI), San Diego, CA 92037, USA; agrifoni@lji.org (A.G.); alex@lji.org (A.S.); dweiskopf@lji.org (D.W.); 7 Division of Infectious Diseases and Global Public Health, Department of Medicine, University of California San Diego, San Diego, CA 92093, USA

**Keywords:** T cells, Zika, congenital Zika syndrome (CZS), Th17 cells, pregnancy

## Abstract

High levels of T helper 17 cell (Th17)-related cytokines have been shown in acute Zika virus (ZIKV) infection. We hypothesized that the high levels of Th17-related cytokines, associated with a regulatory environment during pregnancy, create a favorable milieu for the differentiation of CD4+Th17 cells. We present data from a cross-sectional study on mothers who confirmed ZIKV infection by qRT-PCR and their children. We also recruited non-pregnant women infected with ZIKV in the same period. ZIKV infection occurred between 2015 and 2017. We collected samples for this study between 2018 and 2019, years after the initial infection. We highlight that, after in vitro stimulation with ZIKV CD4 megapool (ZIKV MP), we found a lower frequency of IL-17-producing CD4+ T cells (Th17), especially in the mothers, confirmed by the decrease in IL-17 production in the supernatant. However, a higher frequency of CD4+ IL-17+ IFN-γ+ T cells (Th1Th17) responding to the ZIKV MP was observed in the cells of the mothers and children but not in those of the non-pregnant women. Our data indicate that the priming of CD4 T cells of the Th1Th17 phenotype occurred preferentially in the mothers who gave birth to children with CZS and in the children.

## 1. Introduction

Zika virus (ZIKV) is a flavivirus that was first identified in Uganda in 1947 [1]. The first cases of ZIKV in northeastern Brazil were confirmed in 2015 [2,3], and it spread rapidly between 2015 and 2016 in South and Central America and the Caribbean [4]. The highest number of ZIKV cases were reported in Brazil [5]. In general, most ZIKV-infected individuals are asymptomatic, while a small proportion present with an acute, self-limited exanthematic illness [6]. However, in areas with the confirmed transmission of ZIKV, an unexplained and unexpected increase in cases of neurological abnormalities was reported among babies born to women living in these areas [7]. This causal association between ZIKV infection and fetal malformations, known as congenital Zika syndrome (CZS), was subsequently confirmed in studies that detected ZIKV in amniotic fluid and through evidence that the virus can reach the fetal tissues by crossing the placental barrier [8,9,10,11]. For all these reasons, a state of public health emergency was declared in Brazil.

Knowledge about the immune response to ZIKV in pregnant women and their babies is still limited. The activation of cells and mechanisms of innate immunity, followed by adaptive immunity during viral infection, usually results in eliminating the virus; however, some viruses escape from this response, leading to persistence of the infection, or even, in situations of dysregulation of the immune response, favoring the progression of the disease [12,13]. 

There is some evidence showing that human viruses can initiate the activation of immune system responses, in which CD4 helper T cells (Th) are differentiated into cytokine-secreting effector subpopulations, such as IL-17-producing Th17 cells [14]. Despite their key role in suppressing certain viral infections, Th17 cells are also involved in the induction of detrimental conditions since they can promote tissue damage and mediate chronic inflammation in a wide range of target organs [15]. Th17 cells are a subset of CD4 T cells, and naive CD4 T cell differentiation into effector Th17 cells is dependent on the transcription factor retinoic acid-related (RAR) orphan gamma receptor (RORC2) expression and on the presence of the cytokines IL-6, TGF-β and IL-1β [16,17,18]. Th17 cells are known as the main producers of the cytokine IL-17A but also produce IL-17F, IL-22, IL-26 and CCL20 [16]. Studies have demonstrated that IL-17-producing CD4 T cells express the chemokine receptor CCR6, although not all CD4+ CCR6+ T cells secrete IL-17A [17,19,20]. CCL20, a chemokine that is highly produced by Th17, is the only chemokine ligand for CCR6. The expression of CCR6/CCL20 allows Th17 cell chemotaxis to a wide diversity of tissues, such as the intestine, joints, central nervous system and skin [21,22,23].

Two functionally distinct subpopulations of Th17 cells can be identified based on the expression of CCR4 and CXCR3 in CCR6+ cells. Classically, Th17 cells are identified as CCR6+ CCR4+ CXCR3- cells, secrete IL-17A and express RORC [17]. CCR6+ CCR4- CXCR3+ cells, on the other hand, feature Th1Th17 cells, which produce IL-17A and IFN-γ [17,24,25,26]. Th1Th17 cells present characteristics of both Th1 and Th17 cells since they express the transcription factor RORC and T-bet of Th1 cells [27]. Two other subpopulations of Th17 cells have been characterized. They were identified as CCR6+ double negatives (CCR6+DN; R6+DN; CXCR3- CCR4-) and CCR6+ double positives (CCR6+ DP; R6+DP; CXCR3+ CCR4+). Both subpopulations produce IFN-γ. CCR6+ DN cells express CCR7 and CXCR5, which are related to lymph node chemotaxis, and the high expression of STAT3 and IL-17F mRNA, identified as markers of early-stage differentiation of Th17. The CCR6+ DN cells express genes related to cell survival and proliferation. On the other hand, CCR6+ DP may represent a later stage of differentiation since they express high levels of senescence markers, such as LMNA. Compared with Th17 cells and CCR6+ DP, CCR6+ DN has been proposed as an early stage of differentiation [28].

It has been shown that ZIKV-infected patients have an elevated level of Th17-related cytokines, such as IL-17, IL-1β and IL-6, associated with the function and differentiation of Th17 cells, in comparison with ZIKV-convalescent or healthy individuals [29,30]. In addition, in the brain tissue of infants who died of microcephaly due to ZIKV infection during pregnancy, the presence of an intense inflammatory infiltrate and high levels of inflammatory cytokines such as IL-17 has been shown, compared with babies who died from other causes [31]. However, to date, no study has evaluated the role of Th17 cells in ZIKV infection [29,32].

Pregnancy is characterized by a unique regulated immunological condition, with high levels of TGF-β in systemic circulation and in the placenta [33]. In addition, ZIKV infection induces high levels of IL-17, IL-6 and IL-1β [29,30,32]. Together, these elements could favor an ideal environment for differentiating Th17 cells, even though there are still few studies reporting on the role of Th17 or IL-17 in Zika. 

There are still few studies on the role of Th17 or IL-17 in Zika. As we describe in the discussion below, it is generally agreed that elevated levels of IL-17 and other Th17-related cytokines appear in the acute phase, during viremia [29,32]. Together, these elements could favor an ideal environment for the differentiation of Th17 cells in the acute phase of infection. To address this, we applied ZIKV peptide megapools (ZIKV MP), which were used in in vitro stimulation experiments using PBMCs from these individuals. The aim was to reveal the potential for the differentiation of memory Th17 cells. In addition, considering that the mechanisms that lead to ZIKV-associated microcephaly are not completely understood, our study focused on women who became infected during pregnancy and on children born to them, whether affected by CZS or not.

We used two scenarios to carry out this study: first, ZIKV infection occurred during pregnancy, a period in which a regulatory environment creates a favorable situation for the differentiation of Th17 cells, and, second, authors detected high levels of Th17 cell-related cytokines in acute ZIKV infection. Thus, we assessed whether there is greater differentiation of ZIKV-specific Th17 cells in mothers who had babies with CZS than in mothers who had asymptomatic babies. Likewise, we supposed that Th17 cells occurred preferentially in children born with CZS compared to asymptomatic children. These issues can be assessed years after acute infection using the in vitro restimulation of ZIKV-specific T cells with ZIKV peptides.

## 2. Materials and Methods

### 2.1. Study Design, Volunteers and Samples

A cross-sectional study was performed in mothers who had a rash during pregnancy and who had confirmed Zika (*n* = 22), and children born (*n* = 20) to these mothers, coinciding with the ZIKV outbreak in Brazil (from November 2015 to May 2017). These individuals were recruited in the Exanthematic Diseases Unit at the Hospital Universitário Antonio Pedro of the Universidade Federal Fluminense (HUAP/UFF) located in Niteroi, Rio de Janeiro (Brazil). ZIKV infection during pregnancy was confirmed by a positive quantitative real-time qRT-PCR test on serum and/or urine samples at the flavivirus reference laboratory of Rio de Janeiro State (LACEN, RJ, Brazil) [34]. A positive qRT-PCR result at any point from the maternal rash onset until the first 5 days of rash or until the 14th day for urine samples confirmed the ZIKV infection. Twenty-two pregnant mothers aged 21–45 years who presented a rash, and/or other clinical symptoms suggestive of infection by arbovirus, such as fever, headache, arthralgia, myalgia and conjunctival hyperemia, were included. A positive qRT-PCR for chikungunya or dengue was used as an exclusion criterion. Additionally, mothers with positive test results for syphilis, toxoplasmosis, rubella, cytomegalovirus and/or HIV infection were also excluded from the study (Table 1).

Twenty children aged 17–41 months with a history of intrauterine exposure to ZIKV were divided into two groups. The asymptomatic ZIKV group was composed of children with maternal exposure to ZIKV during pregnancy and did not present clinical evidence of CZS. The CZS ZIKV group consisted of children with exposure to ZIKV during pregnancy who presented evidence of CZS. In both groups, mothers had negative results for other infectious agents. All participants were included in an ongoing clinical follow-up program and were clinically evaluated by a multidisciplinary team [35]. Neuroimaging exams, such as CT (cranial tomography), skull ultrasound and/or MRI (magnetic resonance imaging), were performed to identify radiological alterations compatible with congenital infectious diseases. According to the Ministry of Health of Brazil, the presence of CZS is defined by maternal ZIKV infection confirmed by a qRT-PCR test, accompanied by two or more clinical parameters, such as microcephaly and/or other neurological abnormalities; functional disorders, such as irritability, dysphagia and spasms; visual or auditory anomalies [36]. It was not possible to confirm whether children who were born asymptomatic were directly exposed to ZIKV in utero (Table 1).

As a control group, we recruited six non-pregnant women (women) with confirmed ZIKV infection by qRT-PCR in the same period (2015–2017). This group was similar in age to the group of mothers (Table 1). DENV and chikungunya virus RNA were not detected in any tested individuals.

As mentioned, ZIKV infection occurred between 2015 and 2017. The samples collected for this study were from 2018 and 2019, years after the initial infection. In Table 1, the “illness time” column corresponds to the estimated time in months between the initial acute infection and the moment when the individual’s sample was collected and tested.

Using the Kruskal-Wallis statistical test, women had a longer “illness time” than mothers.

Up to 15 mL of peripheral venous blood from adult patients and up to 8 mL from neonates were collected in tubes with ACD anticoagulant (22.0 g/L sodium citrate, 8.0 g/L citric acid and 24.5 g/dextrose L) (BD Vacutainer ACD Solution A).

### 2.2. Detection of Anti-DENV IgG and Anti-ZIKV IgG Antibodies by an In-House ELISA

Blood donations from these donors were collected at HUAP/UFF from 2018 to 2019. The presence of detectable DENV-specific immunoglobulin G (IgG) titers [37] or ZIKV-specific IgG titers [38] in the serum was considered to determine previous exposure and/or infection to DENV or ZIKV, respectively. ELISA IgG tests specific for ZIKV (Euroimmun, Germany) and specific for DENV (Panbio, Australian) were performed according to the manufacturer’s instructions. 

From the 22 symptomatic mothers with a qRT-PCR positive test result for ZIKV, 13 (59.1%) had both anti-ZIKV IgG and anti-DENV IgG (Table 1).

From the 20 children with a history of intrauterine exposure to ZIKV, we could not perform the anti-DENV IgG and anti-ZIKV IgG assays on two asymptomatic children and one CZS child due to the insufficient sample volume (Table 1).

Regarding the six women infected with ZIKV, all presented both positive anti-ZIKV IgG and anti-DENV IgG serology (Table 1). 

### 2.3. The Plaque Reduction Neutralization Test (PRNT)

The PRNT assay was performed on plasma samples for the detection of ZIKV and DENV-1-neutralizing antibodies. PRNT was not performed for any of the four DENV serotypes since the plasma volume was insufficient for this. In this way, we chose to evaluate DENV-1-neutralizing antibodies since this was the serotype with the highest prevalence in Rio de Janeiro in the period in which the samples were collected (2015–2016) [39].

Briefly, we used the ZIKV/H.sapiens/Brasil/ES2916/2015 strain identified in the State of Espírito Santo, Brazil. A cutoff value of 50% and 90% for PRNT positivity was defined (PRNT_50_ and PRNT_90_). Samples in which we detected neutralizing antibodies for ZIKV were also submitted to PRNT_90_ for dengue virus serotype 1 (DENV-1 from West Pacific). Additionally, the maximum plasma dilution (1:10–1:320) needed to reduce arbovirus plaque formation by 90% among Vero ATCC CCL-81 cells was determined in the assay, following standard protocols [40,41]. Plasma samples were heat-inactivated (56 °C, 30 min) before the neutralization assay. Next, an equal volume of each of the inactivated samples and virus mixture was transferred to a well-containing Vero cell followed by an initial screening at a dilution of 1:10 in 6-well plates at 37 °C for 60 min. The samples that were able to neutralize ZIKV by at least 50 or 90% were tested in posterior assays at serial 2-fold dilutions to determine 50% or 90% endpoint titers.

Regarding DENV-1, plasma samples were considered to have DENV-1-neutralizing antibodies when a plasma dilution of at least ≥10 (1:10) reduced not less than 90% of DENV-1 viral plaque formation.

From the 18 mothers who presented anti-ZIKV IgG, all of them had ZIKV-neutralizing antibodies with PRNT_50_ titer > 320. Regarding the children, we were not able to perform the PRNT assay on seven of them since the sample volume was insufficient. From all of the 13 children, only two asymptomatic children had both detectable anti-ZIKV IgG and anti-DENV IgG, and one of them also presented both ZIKV and DENV-1-neutralizing antibodies. 

Finally, all six women infected with ZIKV had both positive anti-ZIKV IgG and anti-DENV IgG serology. Regarding the PRNT_90_ assays, we could not perform one of them since the sample volume was insufficient. None of them had neutralizing antibodies to ZIKV using PRNT_90_, but four of the five tested had titers using PRNT_50_, and only one did not present DENV-1 neutralizing antibodies (Table 1).

### 2.4. PBMC Isolation

Plasma and peripheral blood mononuclear cells (PBMCs) were isolated by Ficoll-Paque PLUS density gradient centrifugation (GE Healthcare, North Richland Hills, TX, USA) and frozen in fetal bovine serum (FBS, Gibco, Invitrogen Co, Waltham, MA, USA) containing 10% (*vol*/*vol*) dimethyl sulfoxide (DMSO) (Sigma-Aldrich, Burlington, MA, USA). On the day of the experiment, cells were thawed and used directly for the in vitro assays.

### 2.5. ZIKV CD4 MegaPool Description

ZIKV CD4 megapool peptides (ZIKV MP) were designed and validated, as previously described [42,43]. In brief, a consensus sequence was generated by MAFFT alignment after querying the availability of NCBI polyprotein ZIKV sequences and BLAST to a corresponding ZIKV isolate, which was able to represent most of the viral sequence analyzed (ID: 64320) [44]. Then, based on the polyprotein sequence, by using the TepiTool [45] available in the immune epitope database analysis resource (IEDB-AR) [46], CD4 T cell-specific epitopes were predicted. To design the ZIKV CD4 MP, the “7-allele-method” [47] was applied with a cutoff of ≤20. Next, the predicted epitopes were separately clustered for CD4 and CD8 T cells by applying the cluster-break method with a 70% cutoff for sequence identity in the IEDB cluster 2.0 tools [48]. After the bioinformatic analyses, the corresponding peptides were synthesized as a crude material (A&A, San Diego, CA, USA), resuspended in DMSO, and pooled according to CD4 MP composition followed by sequential lyophilization.

The ZIKV MP was designed considering the most frequent HLA allelic variants around the world. In this way, it is possible to capture reactivity independently from geographical location, as previously shown in the context of both ZIKV- and DENV-specific T cell responses [49,50,51,52]. While we cannot exclude the possibility that the specific population considered in our study might express an allelic variant that is not frequent in the worldwide population, the MPs are still designed to provide a 90% or higher worldwide population coverage.

### 2.6. In Vitro T Cell Stimulation

Briefly, peripheral blood mononuclear cells were cultured in triplicate to wells (2 × 10^5^ cells/well) for 6 h in the presence of supplemented medium only (unstimulated) (RPMI-1640, 10% fetal bovine serum, 1% glutamine, 1% penicillin-streptomycin) or with 1 μg/mL ZIKV MPs, and brefeldin A in both situations, followed by incubation at 37 °C, 5% CO2 [53]. As a positive control of in vitro stimulation, we stimulated the PBMCs from all individuals with phorbol myristate acetate and ionomycin (PMA/Ionomycin). Then, cells were recovered, and the staining for flow cytometry was performed. 

Additionally, PBMCs were stimulated in the same conditions for 18 to 20 h, followed by incubation at 37 °C, 5% CO_2_. As a positive control of in vitro stimulation in these assays, we stimulated the PBMCs from all individuals analyzed with phytohemagglutinin (PHA), followed by the staining for flow cytometry experiments.

### 2.7. Extracellular and Intracellular Cytokine Staining for Flow Cytometry

Subsequently, the stimulated PBMCs were stained with the Abs used for the extracellular staining flow cytometry experiments listed in Table S1. For intracellular staining, PBMCs were permeabilized with saponin (0.05%). The intracellular cytokine staining (ICS) was performed in cells from the 6 h stimulation experiments, with anti-IL-17A and anti-IFN-γ antibodies. The intracellular staining of transcription factors with anti-T-bet and anti-GATA-3 antibodies was performed on cells from the 20 h stimulation experiments. The data were collected using a BD FACSAria III flow cytometer and analyzed using FlowJo 10.5.2 software (Tree Star1, Ashland, OR, USA).

### 2.8. Statistical Analysis

Comparisons between the two groups were performed using the non-parametric Mann–Whitney rank sum test (two-tailed analyses). Outcome variables were compared among the groups of study using the Kruskal–Wallis test, followed by Dunn’s multiple comparisons test. The statistical analyses within each donor group were performed using Friedman’s test followed by Dunn’s multiple comparisons test. In addition, the Wilcoxon matched-pairs signed rank test between unstimulated (“−“) and ZIKV MP stimulation was performed in each group of donors. Asterisks indicate significant differences (* *p* < 0.05, ** *p* < 0.01, *** *p* < 0.001, **** *p* < 0.0001) determined using GraphPad PRISM (version 5) (GraphPad Software, San Diego, CA, USA).

### 2.9. Study Approval

This approved study is titled “Clinical follow-up of pregnant women with rash and their children: prospective study cohort”, approval number 56913416.9.0000.5243, 29 March 2017.

## 3. Results

### 3.1. Variability of CD4+ T Cell Phenotypes among Adult Women and Children after In Vitro Stimulation with ZIKV Megapool (ZIKV MP)

**Figure 1:**
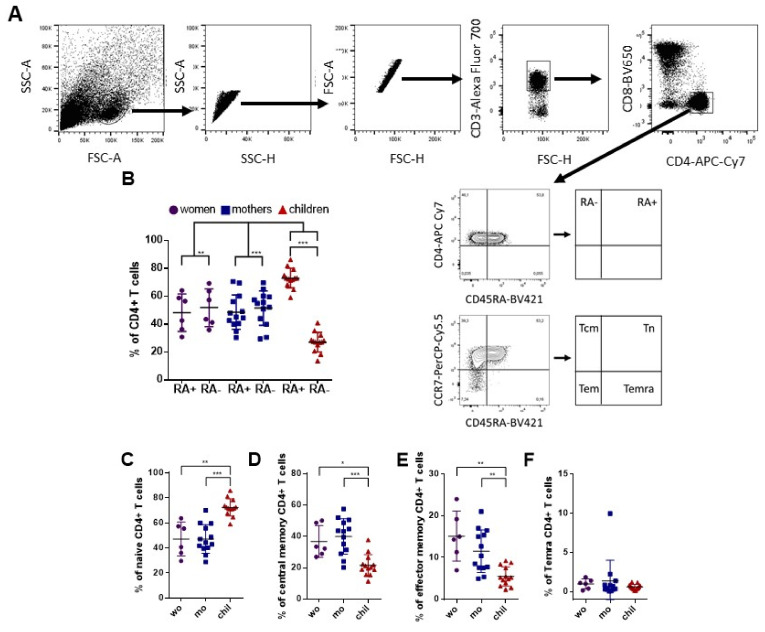
Subpopulations of CD4+ T cell phenotypes among individuals with a history of ZIKV infection. CD4 ZIKV-restricted responses among women (from non-pregnant women infected with ZIKV, violet circles, *n* = 6), mothers (from pregnant mothers infected with ZIKV, blue squares, *n* = 13) and children (from children born to mothers infected with ZIKV during pregnancy, red triangles, *n* = 13) with histories of ZIKV infection, after 6 h of in vitro stimulation with ZIKV MP. (**A**) Gating strategy for the flow cytometry plots identifying ZIKV-specific CD4+ T cells. Black arrows indicate the step-by-step analysis. (**B**) Percentages of CD4+ T cells that express CD45RA (RA+) and that do not express CD45RA (RA–). (**C**) Percentage of naive CD4 T cell subsets (Tn: CD45RA+CCR7+); (**D**) central memory (Tcm: CD45RA–CCR7+); (**E**) effector memory cells (Tem: CD45RA–CCR7–) and (**F**) effector memory RA T cells (Temra: CD45RA+CCR7–). (**B**) Differences between CD45RA+ and CD45RA– CD4+ T cells were analyzed within each group using a Wilcoxon matched-pairs signed rank test. In addition, differences in the frequencies of each RA+ or RA–among the groups were analyzed using the Kruskal–Wallis test followed by Dunn’s multiple comparisons test. (**C**–**F**) Differences in subsets of CD4+ T cells among the groups were analyzed using the Kruskal–Wallis test followed by Dunn’s multiple comparisons test. Data are expressed as the mean with standard deviation for each group. Each data point represents a single individual determination. Asterisks indicate significant differences (* *p* < 0.05, ** *p* < 0.01, *** *p* < 0.001).

We compared the phenotypic characteristics of CD4+ T cells in women (non-pregnant women infected with ZIKV), mothers (pregnant mothers infected with ZIKV) and children (children born to mothers infected with ZIKV during pregnancy). We demonstrated the gating strategy for the flow cytometry plots identifying ZIKV-specific CD4+ T cells in Figure 1A. As noted, children have a prominent population of CD4+ T cells that express CD45RA, while adults have similar frequencies of cells that express and do not express CD45RA+ (Figure 1B). CD45RA+ T cells include naive T (Tn) cells but also effector memory T cells re-expressing CD45RA (Temra) cells. To determine the nature of CD4+ T cells that express CD45RA, we evaluated the proportions of T cells that belong to the Tn (CD45RA+ CCR7+) and Temra (CD45RA+ CCR7−) subsets, as well as the Tcm canonical memory subset (CD45RA− CCR7+) and effector T memory subset (Tem; CD45RA− CCR7−). We then compared the distribution of subsets of cells after in vitro stimulation with ZIKV MP. The children’s CD4+ T cells were predominantly Tn cells (Figure 1C). Tcm cells were the largest subset of CD4+ T cells in adult women, followed by Tem cells (Figure 1D,E). The frequency of Temra cells was below 1% in all groups, and no difference in the Temra cells was observed among the groups (Figure 1F). Thus, there was variability in CD4+ T cell phenotypes in response to in vitro stimulation with ZIKV MP among adult women and children with a history of ZIKV infection. It is possible that this variability is simply due to age. However, it would be very interesting to assess whether infection during pregnancy or vertical transmission could interfere with this observed variability.

### 3.2. Higher Frequency of Memory Th1 Cells in Mothers Compared to Children

**Figure 2:**
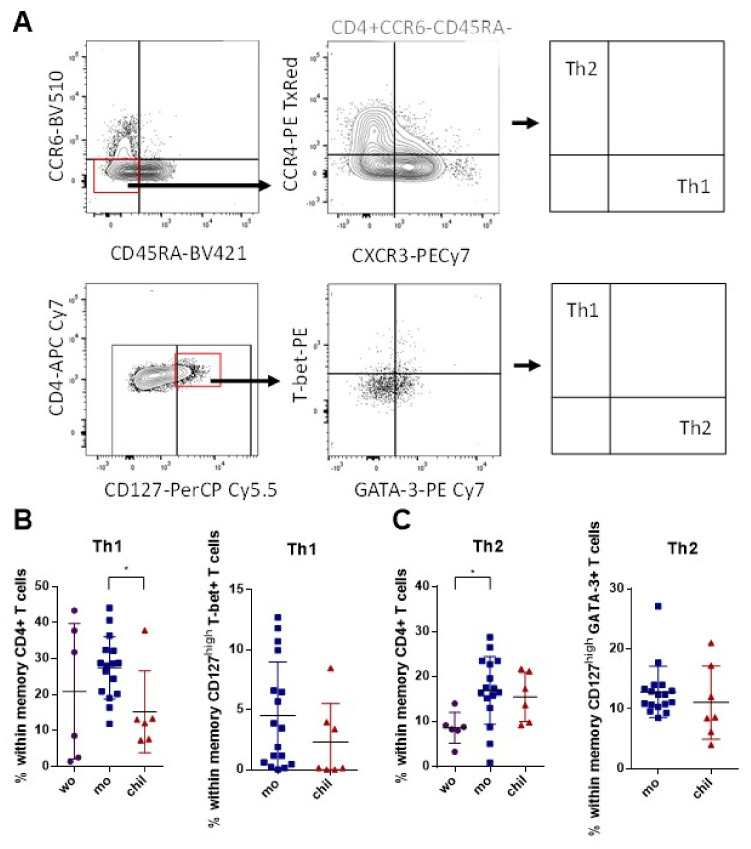
Subpopulations of Th1 and Th2 CD4+ T cell phenotypes among individuals with a history of ZIKV infection. Th1 and Th2 CD4+ subsets were measured in women (from non-pregnant women infected with ZIKV, violet circles, *n* = 6), mothers (from pregnant mothers infected with ZIKV, blue squares, *n* = 16) and children (from children born to mothers infected with ZIKV during pregnancy, red triangles, *n* = 6) with histories of ZIKV infection without stimulation. (**A**) Representative gating of CCR4 and CXCR3 among CD4+ cells that do not express either CCR6 or CD45RA from donor PBMCs is shown. Th1 cells were identified from CCR4− CXCR3+ and Th2 from CCR4+ CXCR3−. In other staining, we gated CD127+ CD4+ T cells with the specific Th1 transcription factor T-bet, and Th2 cells for GATA-3. Black arrows indicate the step-by-step analysis. (**B**) Percentage of Th1 CD4+ cells from surface markers and from transcription factor T-bet among the groups. (**C**) Percentage of Th2 CD4+ cells from surface markers and from transcription factor GATA-3 among the groups. (**B**,**C**) Differences in the frequencies of Th1 and Th2 CD4+ cells from surface markers were analyzed using the Kruskal–Wallis test followed by Dunn’s multiple comparisons test. Furthermore (**B**,**C**), differences in the frequencies of Th1 or Th2 CD4+ cells from intracellular staining of T-bet and GATA-3 were analyzed using the Mann–Whitney test. Data are expressed as the mean with standard deviation for each group. Each data point represents a single individual determination. Asterisks indicate significant differences (* *p* < 0.05).

Previous studies have shown that CCR4 and CXCR3 expression among cells that do not express CCR6 result in functionally distinct subsets of CD4+ memory T cells: Th1 (CCR4− CXCR3+ CCR6−) and Th2 (CCR4+ CXCR3− CCR6−) [17]. Moreover, we combined IL-7 receptor (CD127) surface expression as a marker for long-living memory T cells [54] with the expression of the Th1-specific transcription factor T-bet, and the Th2-specific transcription factor GATA-3. These results were evaluated in cells without in vitro stimulation with ZIKV MP, following the analysis strategy presented in Figure 2A. There was a trend towards a higher frequency of memory Th1 cells identified according to the surface markers in the group of mothers, compared with the group of children. No difference was found between the pregnant and non-pregnant women. These data were confirmed when this analysis was performed in relation to the expression of T-bet (Figure 2B). Regarding the Th2 memory profile, similar frequencies were observed between the groups of mothers and children, regarding characterization according to surface markers. Among the adult women, the group of mothers had a higher frequency of memory Th2 cells than the women. Between the mothers and children, the data regarding the expression of GATA-3 were confirmed (Figure 2C). These data highlight a difference in the frequency of memory Th1 cells between the mothers and children, which was independent of stimulation with ZIKV MP. A differential response profile between the groups could somehow interfere with the immune response to distinct antigens at different stages of life [55,56].

### 3.3. Higher Frequency of Memory Th17 Cells and Lower Frequency of R6+DN in the Mothers and Children, Compared with Women

**Figure 3:**
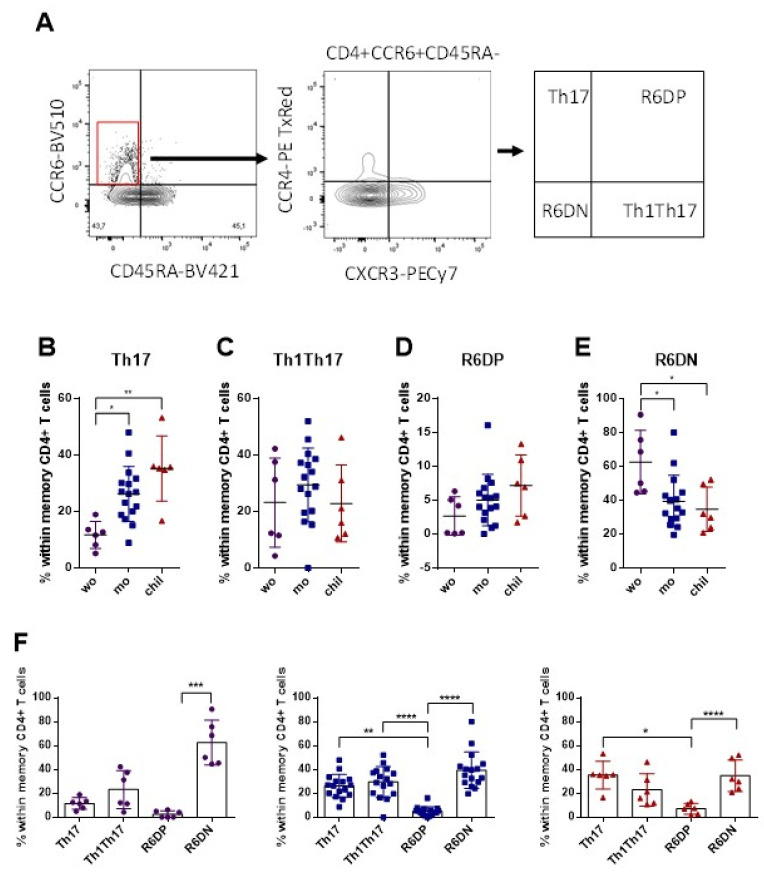
Subpopulations of Th17, Th1Th17, R6+DN and R6+DP CD4+ T cell phenotypes among individuals with a history of ZIKV infection. Th17 CD4+ subsets were measured in women (from non-pregnant women infected with ZIKV, violet circles, *n* = 6), mothers (from pregnant mothers infected with ZIKV, blue squares, *n* = 16) and children (from children born to mothers infected with ZIKV during pregnancy, red triangles, *n* = 6) with histories of ZIKV infection without stimulation. (**A**) Representative gating of CCR4 and CXCR3 among CD4+ cells that express CCR6 but do not express CD45RA from donor PBMCs is shown. Th17 cells were identified from CCR4+ CXCR3−, Th1Th17 from CCR4− CXCR3+, R6+DN from CCR4− CXCR3− and R6+DP from CCR4+ CXCR3+. Black arrows indicate the step-by-step analysis. Percentages of (**B**) Th17 (**C**) Th1Th17, (**D**) R6+DP and (**E**) R6+DN CD4+ cells among the groups. (**F**) Relative contribution of each Th17 subset within each group. (**B**–**F**) Differences in the frequencies of Th17 CD4+ subsets were analyzed using the Kruskal–Wallis test followed by Dunn’s multiple comparisons test. In (**F**), differences in the frequencies of Th17 subsets between the groups were analyzed using the Friedman test followed by Dunn’s multiple comparisons test. Data are expressed as the mean with standard deviation for each group. Each data point represents a single individual determination. Asterisks indicate significant differences (* *p* < 0.05, ** *p* < 0.01, *** *p* < 0.001, **** *p* < 0.0001).

The expression of CCR6 also identified subsets of memory CD4+ T cells: Th17 (CCR4+ CXCR3−), Th1Th17 (CCR4− CXCR3+), R6+DP (CCR4+ CXCR3+) and R6+DN (CCR4− CXCR3−). These results were evaluated in cells without stimulation, with ZIKV MP. The gating strategy is shown in Figure 3A. We observed a higher frequency of Th17 memory cells in the group of children and mothers, compared with the women (Figure 3B). There was no difference in the frequencies of Th1Th17 and R6+DP cells among the groups (Figure 3C,D). On the other hand, we observed a higher frequency of R6+DN memory cells in the group of women than among the mothers, and in relation to the children (Figure 3E). Despite typical group variations in the frequencies of Th17 subsets, the highest frequencies of R6+DN were observed in the group of non-pregnant women, while the frequencies of Th17, Th1Th17 and R6+DN were similar in the groups of mothers and children (Figure 3F). We highlight here a high frequency of memory Th17 cells in mothers and children, while the women had a high frequency of R6+DN cells, which was independent of the stimulation with ZIKV MP.

### 3.4. Lower Frequency of Memory CD4+ CCR6+ Cells after Stimulation with ZIKV MP in Individuals Who Had Recovered from ZIKV Infection

In general, CD4+ T cells that express CCR6 are associated with the Th17 lineage [19]. Although not all CCR6+ T cells produce IL-17, a significant fraction of non-IL-17A-producing CCR6+ T cells become IL-17A producers after specific signals in vitro [57]. We now explore the frequency of memory cells that have long-lasting properties, through the evaluation of CD4+ T cells of the effector memory (TEM)/transitional memory (TM) and central memory (TCM), to assess the potential of these cells for acquiring effector functions after stimulation with ZIKV MP.

**Figure 4:**
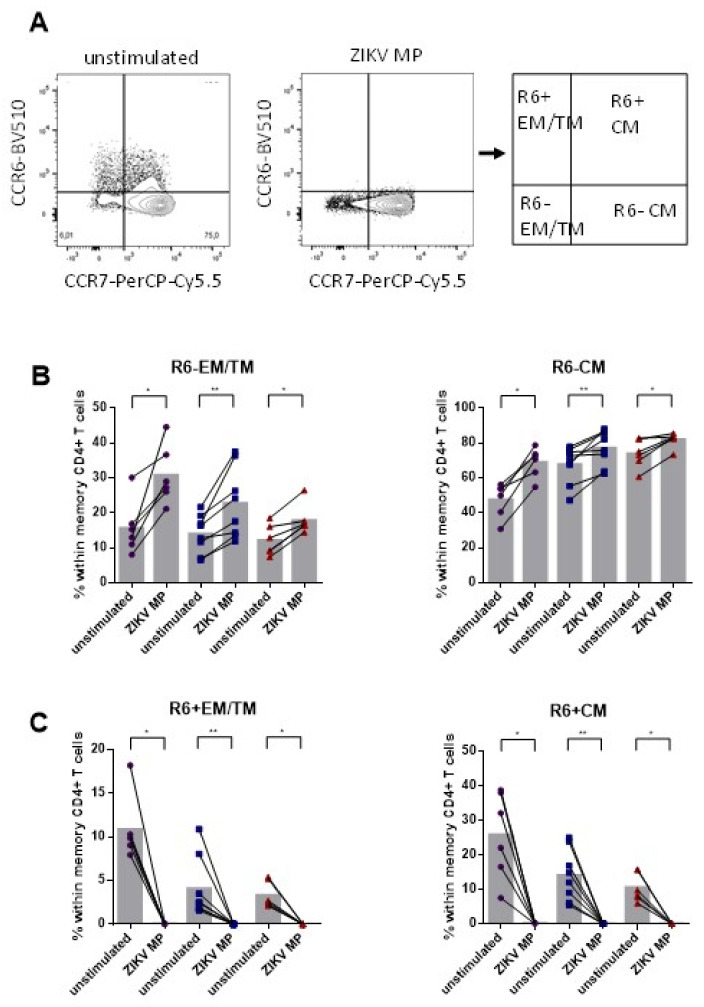
Frequency of memory CD4+ CCR6+ subsets after stimulation with ZIKV MP in individuals with a history of ZIKV infection. Memory CCR6+ and CCR6− CD4+ subsets were measured in women (from non-pregnant women infected with ZIKV, violet circles, *n* = 6), mothers (from pregnant mothers infected with ZIKV, blue squares, *n* = 8) and children (from children born to mothers infected with ZIKV during pregnancy, red triangles, *n* = 6) with histories of ZIKV infection after ZIKV MP stimulation. (**A**) Representative gating of CCR6 among CD4+ cells according to CD45RA− and/or CCR7+ expression from donor PBMCs is shown. Black arrows indicate the step-by-step analysis. In addition, R6− or R6+EM/TM cells were identified through the absence of CCR7 expression and R6− or R6+CM cells expressing CCR7. (**B**) Percentages of R6− EM/TM and R6−CM. (**C**) Percentages of R6+ EM/TM and R6+CM. Differences between cells stimulated with ZIKV MP and unstimulated cells were analyzed using the Wilcoxon matched-pairs signed rank test. Each data point represents a single individual determination without (unstimulated) and after stimulation with ZIKV MP. Grey bars represent the mean for each group. Asterisks indicate significant differences (* *p* < 0.05, ** *p* < 0.01).

The gating strategy is shown in Figure 4A. We observed, in all groups of individuals, that the frequencies of R6− EM/TM (effector memory/transitional memory: CCR7−) and R6− CM (central memory: CCR7+) were higher in cells stimulated with ZIKV MP compared to non-stimulated cells (Figure 4B). On the other hand, the frequencies of R6+ EM/TM and R6+ CM were lower in cells stimulated with ZIKV MP than in non-stimulated cells (Figure 4C). Although we demonstrated that there were notable frequencies of Th17 subset cells in our previous data, the frequencies of all R6+ memory subset cells decreased after stimulation with ZIKV MP. These data may be a first indication that most CCR6+ T cells in individuals with a history of Zika may not produce IL-17 after ZIKV MP in vitro.

### 3.5. Lower Production of IL-17A after Stimulation with ZIKV MP in Mothers Who Had Children with CZS

**Figure 5:**
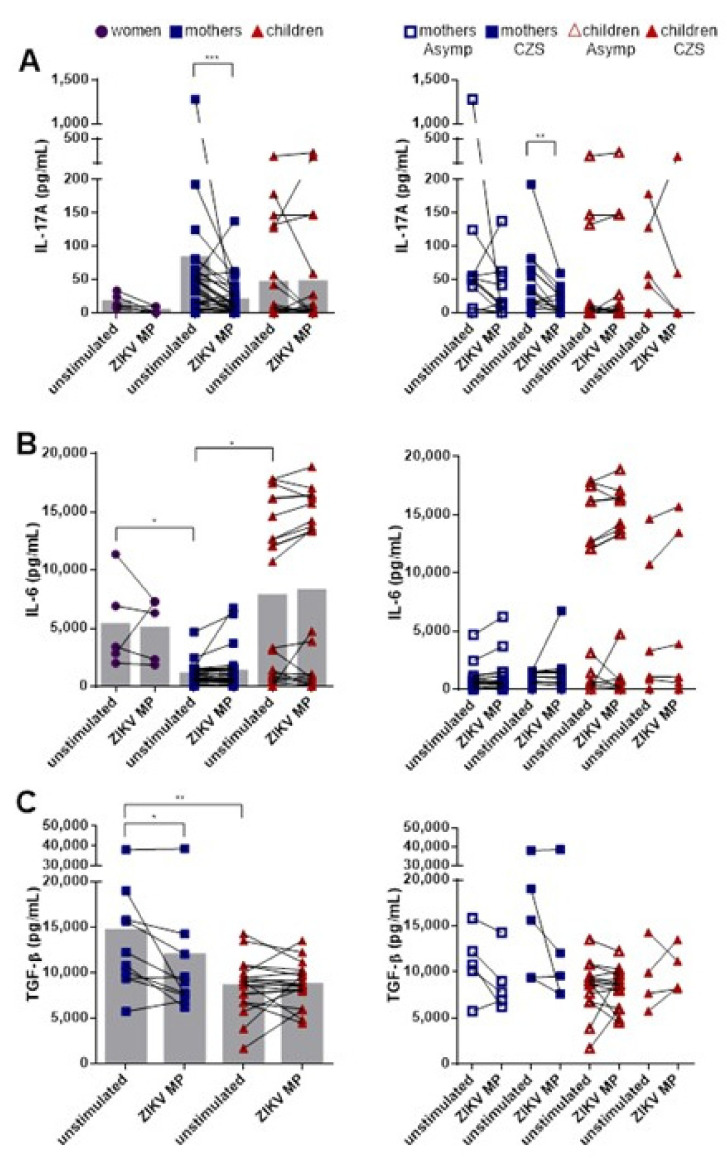
Levels of IL-17A, IL-6 and TGF-β after stimulation with ZIKV MP in individuals with a history of ZIKV infection. Cytokine production in culture supernatants without stimulation or after 20 h of stimulation with ZIKV MP were quantified by means of ELISA. The data represent women (from non-pregnant women infected with ZIKV, violet circles, *n* = 5), mothers (from pregnant mothers infected with ZIKV, blue squares, *n* = 19) and children (from children born to mothers infected with ZIKV during pregnancy, red triangles, *n* = 17) with histories of ZIKV infection. (**A**) IL-17 levels among the groups: between mothers who had asymptomatic babies (*n* = 9) and those who had babies with CZS (*n* = 10), and between asymptomatic children (*n* = 11) and those born with CZS (*n* = 6). Similarly, for (**B**) IL-6 and (**C**) TGF-β. Differences between cells stimulated with ZIKV MP and unstimulated cells were analyzed using the Wilcoxon matched-pairs signed rank test. Each data point represents a single individual determination without (unstimulated) and after stimulation with ZIKV MP. Grey bars represent the mean for each group. Asterisks indicate significant differences (* *p* < 0.05, ** *p* < 0.01, *** *p* < 0.001).

In order to further characterize the potential roles of memory CD4+ CCR6+ T cells in the pathogenesis of ZIKV, PBMC culture supernatants were recovered after stimulation with ZIKV MP and analyzed regarding the production of the cytokines IL-17A, IL-6 and TGF-β, which are related to Th17 cells. Our data initially showed that the baseline levels of IL-17A were not statistically different among the groups, although the groups of mothers and children showed a tendency to have higher IL-17A levels than the women. Stimulation with ZIKV MP decreased the IL-17A levels in the group of mothers, but in the women and children, they remained similar (Figure 5A). The baseline levels of IL-6 in the women and children were higher than those in the mothers. However, stimulation with ZIKV peptides did not alter the IL-6 levels (Figure 5B). High baseline levels of TGF-β were produced by cells in the group of mothers, compared with the children. However, as with IL-17A levels, a reduction in the production of TGF-β was observed after stimulation with ZIKV MP in the group of mothers (Figure 5C). We decided to compare the levels of cytokines between the mothers who had asymptomatic babies and those who had babies with CZS, and between the asymptomatic children and those born with CZS. We observed that the mothers who had children with CZS were those who contributed most to the decrease in IL-17A levels in the presence of ZIKV MP (Figure 5A). No differences were found in relation to the other cytokines (Figure 5B,C). 

Thus, both the mothers and the children tended to have increased IL-17A baseline levels: the children had more IL-6, and the mothers had more TGF-β. These characteristics could all have created a favorable environment for Th17 differentiation. However, after in vitro stimulation with ZIKV MP, our data suggest that Th17 cells were not primed through acute ZIKV infection in these groups. However, it would be interesting to test other non-ZIKV related peptides to evaluate if the decrease in IL-17A secretion is unique to the ZIKV peptides. In addition, other experimental approaches will be needed to confirm this association.

### 3.6. Lower Frequency of Responding IL-17-Producing CD4+ T Cell Subsets and Higher Frequency of Responding IL-17+ IFN-γ+-Coproducing CD4+ T Cell Subsets in Individuals with Histories of ZIKV

**Figure 6:**
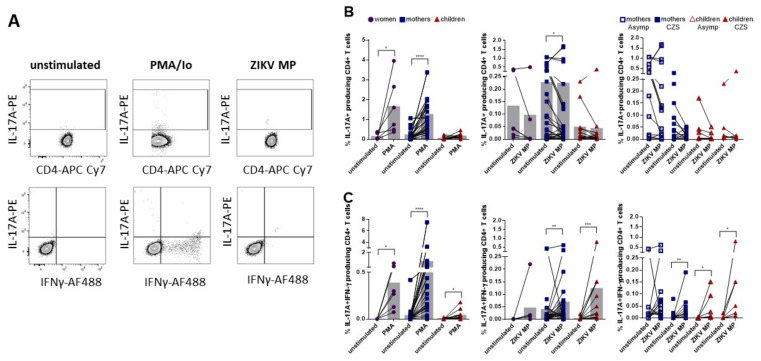
Frequencies of the responding IL-17- and IL-17+ IFN-γ+-producing CD4+ T cell subsets in individuals with histories of ZIKV. Cytokine-producing CD4+ T cells were measured in women (from non-pregnant women infected with ZIKV, violet circles, *n* = 6), mothers (from pregnant mothers infected with ZIKV, blue squares, *n* = 21) and children (from children born to mothers infected with ZIKV during pregnancy, red triangles, *n* = 12) with histories of ZIKV infection, after stimulation with ZIKV MP. (**A**) Representative gating of IL-17+ IFN-γ− and IL-17+ IFN-γ+-producing CD4+ T cell subsets from donor PBMCs is shown. Percentages of (**B**) IL-17+ IFN-γ− and (**C**) IL-17+ IFN-γ+-producing CD4+ T cell subsets in each group. Cytokine-producing CD4+ T cells were also evaluated between the mothers who had asymptomatic babies (*n* = 11) and those who had babies with CZS (*n* = 10) and between the asymptomatic children (*n* = 6) and those born with CZS (*n* = 6). Differences between cells stimulated with ZIKV MP and unstimulated cells were analyzed using the Wilcoxon matched-pairs signed rank test. Each data point represents a single individual determination without (unstimulated) and after stimulation with ZIKV MP. Grey bars represent the mean for each group. Asterisks indicate significant differences (* *p* < 0.05, ** *p* < 0.01, *** *p* < 0.001, **** *p* < 0.0001).

Through using stimulation with ZIKV MP and intracellular cytokine staining (ICS) assays (Figure 6A), we were able to determine the responding IL-17-producing CD4+ T cell subsets. All the individual cells with histories of ZIKV infection were stimulated using the polyclonal stimulus PMA and ionomycin. Apart from children, the remaining individual cells showed the ability to produce IL-17A. After stimulation with ZIKV MP, it was only in the group of mothers that the frequency of IL-17-producing CD4+ T cells decreased in relation to the unstimulated condition, in agreement with the data obtained from the supernatant. No difference was observed in relation to the other groups after in vitro stimulation with ZIKV MP (Figure 6B). In addition to IL-17-producing CD4+ T cell subsets, we were able to determine the responding IL-17+IFN-γ+-coproducing CD4+ T cell subsets (Figure 6A). All cells from individuals were stimulated using polyclonal PMA plus ionomycin, thus confirming the ability of these individuals’ cells to produce both IL-17A and IFN-γ. The most impressive observations were that stimulation with ZIKV MP led to an increase in the frequencies of IL-17A+IFN-γ+ CD4 T cells in the groups of mothers and children but not among the women (Figure 6C). Next, we compared the frequencies of IL-17A+ and IL-17A+IFN-γ+ CD4 T cells between the mothers who had asymptomatic babies and those who had babies with CZS and between the asymptomatic children and those born with CZS. We observed that the mothers who had children with CZS were those who contributed most to the decrease in IL-17-producing CD4+ T cell subsets in the presence of ZIKV MP (Figure 6B). On the other hand, stimulation with ZIKV MP led to increases in the frequencies of IL-17A+ IFN-γ+ CD4 T cells in the mothers who had babies with CZS and in both groups of children (Figure 6C).

Thus, after in vitro stimulation with ZIKV MP, our data suggest that IL-17-producing CD4+ T cell subsets appeared not to be primed through acute ZIKV infection. However, ZIKV MP-responding IL-17+ IFN-γ+-coproducing CD4+ T cell subsets were primed mainly in the mothers and children, but not in the women with histories of ZIKV infection.

## 4. Discussion

Th17 cells have been demonstrated to play a critical role against certain viral infections. Depending on the virus, Th17 cells and Th17-related cytokines can increase antigen-presenting cells’ efficiency, CD8+ T cell cytotoxicity activity and the activity of B cells with the production of neutralizing antibodies and others [58,59,60]. However, Th17 cells are also concerned in mediating tissue damage and orchestrating chronic tissue inflammation in diverse target organs [61]. Furthermore, there are non-pathogenic Th17 cells that also modulate immune responses since they can produce immunosuppressive mediators, such as IL-10 [62]. Understanding the role of Th17 in viral infections could help to predict clinical outcomes and even improve patient treatment since the blockade of the cytokine IL-17 or IL-17-induced pathways represents an important new therapeutic approach towards viral diseases [63,64]. 

We did not use ZIKV-naive individuals as control groups as we demonstrated that memory T cells from ZIKV-seropositive individuals could recognize DENV-derived peptides [53]. This group could generate a discussion about the cross-reactivity between ZIKV and DENV, which is not the purpose of this study.

Studies on the influence of antibodies specific to DENV on the vertical transmission of ZIKV, resulting in congenital and neurological abnormalities in offspring, are still very discordant. Some studies have already confirmed that CZS does not appear to be associated with the severity of the maternal disease, the ZIKV-RNA load at the time of infection [65] or the existence of previous dengue antibodies [65,66]. In contrast, Robbiani et al. showed that serum from the ZIKV-infected pregnant women of the microcephaly cases correlated with higher antibody-enhancing activity titers than that observed among non-microcephaly controls. This supported the hypothesis that an antibody-mediated mechanism could be involved in the pathogenesis of fetal brain injury and microcephaly in humans, which was validated in nonhuman primate models [67].

From our serological data, 70% of the mothers who had asymptomatic children and 50% of the women who had children with CZS had both anti-ZIKV IgG and anti-DENV IgG. The same was true for our PRNT_90_ data since 90% of the mothers who had asymptomatic children and 58% of the mothers who had children with CZS had neutralizing antibodies against ZIKV and DENV-1. It was not our intention here to move forward in the discussion on the relationship between previous DENV infection and cases of CZS, but from the matters discussed in the articles mentioned above, this is an important element that could influence the appearance of cases of CZS. However, based on the detection of anti-DENV IgG and anti-ZIKV IgG and the PRNT titers of DENV-1 and ZIKV in our cohort, we cannot confirm that the presence of anti-DENV IgG is responsible for the appearance of CZS. Unfortunately, we did not have matched samples acquired in the acute phase of infection for comparison purposes, which does not compromise the study’s objectives.

In our study, the initial proposal was to reveal the potential for the differentiation of memory Th17 cells. To this aim, we began by assessing the status and frequency of different CD4+ T cell profiles.

The expression of CD45 isoforms and CCR7 has been extensively studied in peripheral blood T cells in order to assess the status and frequency of T cell profiles. 

Thus, in fact, throughout an individual’s lifetime, the levels of different T cell subpopulations undergo dynamic changes. Thus far, it remains unknown whether ZIKV infection interferes in these changes. It would be interesting to monitor these individuals to assess whether variations in these patterns change or continue over time. 

However, another important analysis is to assess the functional capacity of these cells. Based on the analysis of IFN-γ production among the subsets of CD4+ T cells in our recent publication, using the same cohort of individuals, we observed that only Temra cells, the infrequent ones, are the main producers of IFN-γ for epitopes of ZIKV in women, mothers and children [68]. Thus, in addition to the frequency of CD45 isoform markers, it is important to assess the functional capacity of these cells.

In 2016, Wacleche et al. were the first to reveal the existence of the CCR6+ DN and CCR6+ DP cell subsets, which share functional characteristics with Th17 and Th1Th17. According to these authors, the molecular signature of CCR6+ DN suggested that they represent an early stage of Th17 differentiation. This subset was found to be the most prevalent subset in the blood and lymph nodes of HIV-infected individuals and was able to carry replicative HIV reservoirs in individuals treated with ART [28]. Here, we demonstrated high frequencies of CCR6+ DN (R6+DN) in the group of women, compared with the other groups. Thus, it is possible that in adult women who become infected with ZIKV outside of pregnancy, the priming and differentiating of naive CD4+ T cells will generate Th17 cells with a CCR6+ DN phenotype. More experiments need to be carried out to confirm this hypothesis. 

Furthermore, regarding CCR6, this receptor regulates cell migration in several anatomical sites [69]. CD4+CCR6+ T cells are usually associated with the Th17 lineage [19]. Although not necessarily all CCR6+ T cells can produce IL-17, a significant fraction become IL-17A producers after specific signals in vitro [57]. Thus, according to the Th17 polarization model, there are two independent steps. The first is to acquire the marker of the cell line, and the second is to acquire the effector functions related to that line after antigenic stimulation [70]. Therefore, we explored the frequency of memory cells that have long-lasting properties, through evaluating CD4+ T cells of the effector memory (TEM)/transitional memory (TM) and central memory (TCM), to assess the potential of these cells to acquire effector functions after stimulation with ZIKV MP. Although we demonstrated a notable frequency of cells from the Th17 subset in our previous data, the levels of CCR6+ memory cells decreased after stimulation with ZIKV MP. These data could be a first indication that most CCR6+ T cells may not be committed to IL-17 production after in vitro stimulation with ZIKV MP. This is based on no or little secretion of IL-17 in the supernatant or the decrease in the frequency of IL-17-producing CD4 T cells by ICS assays. However, other experimental approaches must be taken to confirm these associations.

Following on from our discussion of these differential profiles, we now move to the functional analysis and evaluation of our initial objective, which was to assess the different subpopulations of Th17 in response to ZIKV MP. Thus far, all published studies agree that, especially in the acute phase of ZIKV infection, an environment favorable to the activation and differentiation of naive CD4+ T cells into Th17 cells exists. This was demonstrated, for example, in the study by Tappe et al., in which it was shown that acute ZIKV-infected patients showed increased levels of cytokines, related to the differentiation of Th17 cells, such as IL-1β and IL-6 and their effector function, such as IL-17, when compared with those same individuals in the convalescence phase and healthy individuals [29]. In addition, Naveca et al. showed a bimodal variation of viremia during ZIKV infection, with a first peak during systemic disease followed by a peak associated with the presence of the virus in tissues and organs. Their analysis of the biomarker network demonstrated that different dynamics occurred concomitantly with the profiles of bimodal viremia. Both IL-17 and other cytokines exhibited bimodal distribution that accompanied the viremia. According to the authors, this robust response was associated with blood–brain barrier permeability and neuroinvasiveness regarding other flavivirus infections. They demonstrated that there was the involvement of IL-17 and related cytokines, although they highlighted CXCL10 as a biomarker of acute ZIKV infection and a potential target for therapeutic intervention [71]. Moreover, Fares-Gusmao et al. sought to identify unique characteristics for dengue virus (DENV), West Nile (WNV) and ZIKV infections by quantifying soluble immunological markers. Among other characteristics, an increase in IL-17, even in the absence of symptoms, was found in all flavivirus groups, in comparison with uninfected individuals [32].

Although we did not quantify the serum or plasma levels of IL-17 and cytokines in relation to the differentiation of Th17 cells in our three groups of individuals who had recovered from ZIKV infection, we measured the cytokines in cell culture supernatants from these individuals under conditions of with and without (baseline) stimulation with ZIKV MP. Interestingly, the mothers and children tended to have high baseline levels of IL-17A, while the children had more IL-6 and the mothers had more TGF-β, compared with the other groups. Therefore, our data also indicate that the environment was favorable for Th17 differentiation. However, after in vitro stimulation with ZIKV MP, there was a decrease in IL-17A levels in cells, mostly in the group of mothers who had babies with CZS, compared with the unstimulated condition. Since the ZIKV MP was specific for CD4 T cells, we believe that this reduction was related mainly to the activity of these cells. To understand this phenomenon, we also quantified the levels of IL-6 and TGF-β after stimulation by ZIKV MP. Interestingly, TGF-β levels were also reduced in the presence of ZIKV MP, although no difference was observed regarding IL-6 levels. The literature is still quite controversial regarding the need for TGF-β in the differentiation of Th17 in human studies. Several groups have demonstrated that a combination of IL-6, IL-1β and IL-23 is sufficient for the development of human Th17 cells [72,73] and that TGF-β is not essential [74,75], while other researchers reported that TGF-β was essential for human Th17 differentiation [76,77], especially for the induction of T cells homogeneously producing IL-17A. In fact, the absence of TGF-β seems to lead to the generation of Th1Th17 [77]. 

In a study similar to ours, Pereira-Neto et al. evaluated the multifunctionality of T cells from individuals 2 years after ZIKV infection. They stimulated the PBMCs from these individuals with a wide range of synthetic peptides from the entire ZIKV polyprotein, divided into pools according to each of the viral protein’s region. They showed that IFN-γ+ IL-17A+ and IL-17A+ IL-10+T cells could also produce TNF in response to stimulation with antigens of the capsid, prM, NS1 or NS3 regions [78].

In our results, after in vitro stimulation with ZIKV MP, a lower frequency of IL-17-producing CD4+ T cells was found, especially in the mothers, thus confirming the decrease in IL-17 production that was found in the supernatant of this same group. However, a higher frequency of CD4+ IL-17+ IFN-γ+ T cells responding to the ZIKV MP was observed in the cells of the mothers and children, but not in those of the women. Since pregnancy is a unique modulated immunological condition, with high levels of TGF-β in the circulation and in the placenta, and since, as mentioned before, ZIKV infection induces high circulating levels of IL-6 and IL-1β, we can suggest that the priming of CD4 T cells of the Th1Th17 phenotype may have happened during the mothers’ pregnancies, especially among the mothers who gave birth to children with CZS and among their children, regardless of clinical impairment. 

More commonly, the authors use polyclonal stimuli to assess the secretion or intracellular labeling of IL-17, precisely due to the fact that cytokine production is extremely low, making the data unreliable. Recently, De Biasi et al. demonstrated IL-17-producing CD4 cells in patients with COVID-19, using polyclonal stimulation, of less than 1% and in healthy donors, of less than 0.5% [79]. Here, we used the PMA/Ionomycin stimuli to show that we can detect IL-17 by ICS. Assessing IL-17 production after stimulation with ZIKV peptides was not an easy task, mainly due to the fact that the stimulus is targeted to some specific T cell clones. However, we note that the ICS data are partially reproduced in the quantification of IL-17 in the supernatant by ELISA. 

Few studies have addressed differences among mothers who gave birth to children during ZIKV infection. We believe that one of the key issues involving all congenital infections and, in our case, ZIKV infection, is whether vertical transmission in children can cause the progressive impairment of humoral and cellular immunity to different pathogens, which would cause enormous damage to these children. Few current studies address this issue, and it was necessary to refer to studies published many years ago to understand the issue further, in the light of our data.

From the set of data presented, we highlight a preferential profile of Th17 cells in mothers with a history of ZIKV infection during pregnancy and in children exposed vertically. On the other hand, in women with a history of ZIKV infection, CCR6+ DN cells were the favored subpopulation. Interestingly, after stimulation with ZIKV MP, the frequency of CCR6+ memory cells decreased in all groups of individuals. These data may suggest that, even when present, the different subpopulations of Th17 cells in individuals with a history of Zika may not be compromised through the production of the cytokine IL-17A. We also observed that in vitro stimulation with ZIKV MP also led to a decrease in the frequency of IL-17A-producing CD4+ T cells (Th17) in the mothers, with no difference in the other groups. However, surprisingly, we detected an increase in IL-17A+ IFN-γ+-producing CD4+ T cells (Th1Th17 cells) in the mothers and children, but not in the women with a history of ZIKV infection. However, other experimental approaches will be needed to confirm this association.

Some limitations should be considered when interpreting our findings: First, a longitudinal study would be ideal for assessing the profile of ZIKV-specific Th17 cells over time. Second, it was not possible to diagnose ZIKV by RT-PCR in newborns, so we cannot say that the asymptomatic children were infected. Third, we do not exclude the possibility of cross-reactivity for data obtained from commercial ELISA serology kits. Additional serological tests need to be performed to determine the specificity of our patients’ anti-ZIKV and anti-DENV antibodies. Fourth, we cannot exclude the possibility that exposure to DENV, or Chikungunya virus and yellow fever vaccination, distorted or altered T cell responses to ZIKV peptides. Finally, it is indisputable that the inclusion of other control groups in our study could bring greater reliability to the conclusions of our research.

When evaluating individuals who had ZIKV infection years ago, we understand that our data do not directly contribute to the understanding of the pathogenesis of CZS. However, we can extrapolate our findings based on the premise that circulating CD4 T cells evolve from differentiated effector CD4 T cell subsets in the acute phase of the disease [80,81]. For this, we could use methodologies such as experimental approaches that assess specific memory T cells in individuals exposed to DENV or ZIKV [49,68,82,83,84]. In the literature, when Th17 cells acquire a Th1 profile [85], they are characterized by pathogenic cells, mainly in autoimmune diseases [86]. It appears that Th17 cells can enter the central nervous system to carry out neuropathogenic properties previously attributed to Th1 cells [87,88,89]. Our data do not define a clear role for Th1Th17 priming in the mothers of children with CZS. However, we encourage further investigation into the possible implications of Th1Th17 cells for Zika neuropathogenesis in the acute phase of human ZIKV infection and experimental models.

## Figures and Tables

**Table 1:** Characteristics of the recovered mothers infected with Zika virus during pregnancy and their intrauterine exposed children recruited from 2018 to 2019.

Group	Outcome at Birth	ID	Age ^a,b^	Illness Tine ^b^	Gestational Trimester at Onset Rash	RT-qPCR ZIKV	ZIKV Anti-IgG	DENV Anti-IgG	PRNT_50_ ZIKV	PRNT_90_ ZIKV	PRNT_90_ DENV-1
Women		W1	40	36		Pos	Pos	Pos	80, Pos	<10, Neg	≥10, Pos
		W2	40	40		Pos	Pos	Pos	≥320, Pos	<10, Neg	<10, Neg
		W3	23	38		Pos	Pos	Pos	80, Pos	<10, Neg	≥10, Pos
		W4	25	42		Pos	Pos	Pos	40, Pos	<10, Neg	≥10, Pos
		W5	27	35		Pos	Pos	Pos	<10, Neg	<10, Neg	≥10, Pos
		W6	35	36		Pos	Pos	Pos	-	-	-
			**31**	**37**		**6/6 pos**	**6/6 pos**	**6/6 por**	**4/5 pos**	**0/5 pos**	**4/5 pos**
			**(23–40)**	**(35–42)**		**100%**	**100%**	**100%**	**80%**	**0%**	**80%**
Mothers	Asympt.	M1	22	23	3rd	Pos	Pos	Neg	≥320, Pos	160, Pos	<10, Neg
		M2	36	25	3rd	Pos	Pos	Pos	≥320, Pos	≥320, Pos	≥10, Pos
		M3	22	23	1st	Pos	Pos	Neg	≥320, Pos	≥320, Pos	≥10, Pos
		M4	37	23	2nd	Pos	Pos	Pos	≥320, Pos	≥320, Pos	≥10, Pos
		M5	27	22	2nd	Pos	Pos	Neg	≥320, Pos	≥320, Pos	≥10, Pos
		M6	37	24	2nd	Pos	Pos	Pos	≥320, Pos	≥320, Pos	≥10, Pos
		M7	29	23	2nd	Pos	Pos	Pos	≥320, Pos	≥320, Pos	≥10, Pos
		M8	21	23	2nd	Pos	Pos	Pos	≥320, Pos	≥320, Pos	≥10, Pos
		M9	32	40	2nd	Pos	Pos	Pos	≥320, Pos	≥320, Pos	≥10, Pos
		M10	30	35	2nd	Pos	Pos	Pos	≥320, Pos	≥320, Pos	≥10, Pos
			**29**	**26**		**10/10 pos**	**10/10 pos**	**7/10 pos**	**10/10 pos**	**10/10 pos**	**9/10 pos**
			**(21–37)**	**(22–40)**		**100%**	**100%**	**70%**	**100%**	**100%**	**100%**
	CZS	M1	21	19	1st	Pos	Pos	Pos	≥320, Pos	≥320, Pos	≥10, Pos
		M2	23	10	2nd	Pos	Neg	Neg	≥320, Pos	≥320, Pos	<10, Neg
		M3	24	22	1st	Pos	Pos	Neg	≥320, Pos	≥320, Pos	<10, Neg
		M4	42	20	2nd	Pos	Pos	Pos	≥320, Pos	≥320, Pos	≥10, Pos
		M5	25	37	3rd	Pos	Pos	Neg	≥320, Pos	≥320, Pos	≥10, Pos
		M6	40	39	2nd	Pos	Pos	Pos	≥320, Pos	≥320, Pos	≥10, Pos
		M7	45	24	3rd	Pos	Neg	Neg	≥320, Pos	160, Pos	<10, Neg
		M8	41	29	2nd	Pos	Pos	Pos	≥320, Pos	≥320, Pos	<10, Neg
		M9	28	26	2nd	Pos	Pos	Pos	≥320, Pos	≥320, Pos	≥10, Pos
		M10	21	36	before	Pos	Neg	Neg	≥320, Pos	≥320, Pos	<10, Neg
		M11	28	24	1st	Pos	Neg	Pos	≥320, Pos	≥320, Pos	≥10, Pos
		M12	33	35	2nd	Pos	Pos	Pos	≥320, Pos	≥320, Pos	≥10, Pos
			**31**	**28**		**12/12 pos**	**8/12 pos**	**7/12 pos**	**12/12 pos**	**12/12 pos**	**7/12 pos**
			**(21–45)**	**(10–39)**		**100%**	**67%**	**58%**	**100%**	**100%**	**58%**
Children	Asympt.	C1	31	35	x	x	Neg	Pos			
		C2	29	37	x	x	Neg	Neg	<10, Neg	<10, Neg	≥10, Pos
		C3	34	36	x	x	Pos	Pos	≥320, Pos	≥320, Pos	≥10, Pos
		C4	30	36	x	x	Neg	Neg			
		C5	41	45	x	x	Neg	Neg	<10, Neg	<10, Neg	≥10, Pos
		C6	31	37	x	x	-	-			
		C7	32	35	x	x	Neg	Neg	<10, Neg	<10, Neg	<10, Neg
		C8	36	42	x	x	-	-			
		C9	30	36	x	x	Pos	Pos			
		C10	38	42	x	x	Neg	Neg	<10, Neg	<10, Neg	<10, Neg
		C11	24	33	x	x	Neg	Neg	<10, Neg	<10, Neg	≥10, Pos
			**32**	**38**			**2/9 pos**	**3/9 pos**	**1/6 pos**	**1/7 pos**	**4/6 pos**
			**(24–41)**	**(33–45)**			**22%**	**33%**	**14%**	**17%**	**67%**
	CZS	C1	17	18	x	x	Neg	Pos	<10, Neg	<10, Neg	≥10, Pos
		C2	32	36	x	x	Neg	Neg			
		C3	22	28	x	x	Neg	Pos	≥320, Pos	160, Pos	≥10, Pos
		C4	30	36	x	x	-	-	<10, Neg	<10, Neg	≥10, Pos
		C5	32	36	x	x	Neg	Neg	<10, Neg	<10, Neg	≥10, Pos
		C6	24	36	x	x	Neg	Neg	<10, Neg	<10, Neg	≥10, Pos
		C7	27	34	x	x	Neg	Pos	<10, Neg	<10, Neg	<10, Neg
		C8	32	38	x	x	Neg	Pos	<10, Neg	<10, Neg	≥10, Pos
		C9	26	32	x	x	Neg	Neg			
			**27**	**33**			**0/8 pos**	**4/8 pos**	**1/7 pos**	**1/7 pos**	**6/7 pos**
			**(17–32)**	**(18–38)**			**0%**	**50%**	**17%**	**17%**	**86%**

Age ^a,b^, where a—years, b—months. Illness time ^b^, months. In bold: the group results’ median (minimum-maximum) and frequency (%).

## Supplementary Materials

The following are available online at https://www.mdpi.com/article/10.3390/v14020250/s1, Table S1: Antibodies used in BD FACS ARIA IIu Flow Cytometer.
