# Supplementary material for: Involvement of Th1Th17 Cell Subpopulations in the Immune Responses of Mothers Who Gave Birth to Children with Congenital Zika Syndrome (CZS)
Source: Viruses. 2022 Jan 26;14(2):250. doi: 10.3390/v14020250 (PMC8879837; doi:10.3390/v14020250)
Supplement: Supplementary file 1 [file viruses-14-00250-s001.zip › viruses-1477927-supplementary.pdf]

Table S1. Antibodies used in BD FACS ARIA IIu Flow Cytometer.

Target Molecule	Fluorochrome	Clone	Company	Catalog #
IFN-γ	FITC	4S.B3	eBioscience	11-7319-82
IL-17A	PE	BL168	BioLegend	512306
CCR4	PE-CF594	1G1	BD	565391
CCR7	PerCP Cy5.5	G043H7	BioLegend	353220
CXCR3	PECy7	1C6	BD	560831
CD3	AlexaFluor 700	UCHT1	BD	557943
CD4	APCef780	RPA-T4	eBioscience	47004942
CD45RA	eF450	HI100	eBioscience	48045842
CCR6	BV510	11A9	BD	563241
T-bet	PE	4B10	BD	561265
CD127	PerCP Cy5.5	A019D5	BioLegend	351322
GATA 3	PECy7	L50-823	BD	560405
